# Immune-Regulatory and Molecular Effects of Antidepressants on the Inflamed Human Keratinocyte HaCaT Cell Line

**DOI:** 10.1007/s12640-021-00367-5

**Published:** 2021-05-04

**Authors:** Curzytek K., Maes M., Kubera M.

**Affiliations:** 1 Department of Experimental Neuroendocrinology, Maj Institute of Pharmacology Polish Academy of Sciences, Kraków, Poland; 2 Department of Psychiatry, Faculty of Medicine, King Chulalongkorn Memorial Hospital, Bangkok, Thailand; 3 Department of Psychiatry, Medical University of Plovdiv, Plovdiv, Bulgaria; 4 IMPACT Strategic Research Centre, Deakin University, PO Box 281, Geelong, VIC 3220 Australia

**Keywords:** Antidepressant drugs, Contact hypersensitivity, Depression, Cytokines, Adhesion molecules

## Abstract

Allergic contact dermatitis (ACD) is a T cell-mediated type of skin inflammation resulting from contact hypersensitivity (CHS) to antigens. There is strong comorbidity between ACD and major depression. Keratinocytes release immunomodulatory mediators including pro-inflammatory cytokines and chemokines, which modulate skin inflammation and are crucial cell type for the development of CHS. Our previous studies showed that fluoxetine and desipramine were effective in suppressing CHS in different mouse strains. However, the immune and molecular mechanisms underlying this effect remain to be explored. The aim of the current study was to determine the immune and molecular mechanisms of action of antidepressant drugs engaged in the inhibition of CHS response in the stimulated keratinocyte HaCaT cell line. The results show that LPS, TNF-α/IFN-γ, and DNFB stimulate HaCaT cells to produce large amounts of pro-inflammatory factors including IL-1β, IL-6, CCL2, and CXCL8. HaCaT stimulation was associated with increased expression of ICAM-1, a cell adhesion molecule, and decreased expression of E-cadherin. Imipramine, desipramine, and fluoxetine suppress the production of IL-1β, CCL2, as well as the expression of ICAM-1. LPS and TNF-α/IFN-γ activate p-38 kinase, but antidepressants do not regulate this pathway. LPS decreases E-cadherin protein expression and fluoxetine normalizes these effects. In summary, the antidepressant drugs examined in this study attenuate the stimulated secretion of pro-inflammatory cytokines, chemokines, and modulate adhesion molecule expression by the HaCaT cell line. Therefore, antidepressants may have some clinical efficacy in patients with ACD and patients with comorbid depression and contact allergy.

## Introduction

In humans, the progressive industrialization and the associated development of the chemical industry have increased the exposure to a large variety of chemicals. Some of these may function as haptens of low molecular weight that are capable of inducing delayed-type hypersensitivity (DTH), which is an immune response that develops at the site of hapten exposure and is a classical form of T cell-mediated immunity (Blauvelt et al. 2003). Allergic contact dermatitis (ACD) is the most common type of DTH in humans. Contact dermatitis (CD) affects approximately 20% of the general population, whereas occupational CD constitutes up to 30% of all occupational diseases (Thyssen et al. 2007; Diepgen and Weisshaar 2007). The number of etiologic CD factors is very high and is still growing with over 3700 haptens being identified (Martin et al. 2004). The ever-growing number of patients suffering from this disease adversely affects patients’ productivity and health-related quality of life. In contact hypersensitivity responses, there are two phases: an induction phase after the priming contact with a hapten and an elicitation phase that develops after re-exposure to the hapten (Kimber and Dearman 2002). A wide variety of cells (dendritic cells, endothelial cells, mastocytes, keratinocytes, melanocytes, monocytes, neutrophils, and antigen-specific T lymphocytes) and pro-inflammatory factors are involved in both stages (Majewska and Szczepanik 2009).

The skin is the largest organ of the body with an area of approx. 2 m^2^ representing 15% of total body weight (Dąbrowska et al. 2018). The skin barrier comprises local skin cells, including keratinocytes, mast cells, and immune cells, and is, in fact, a component of the immune system (Blauvelt et al. 2003). There is a tight association between the skin and the nervous system which is formed as early as in utero since both organs develop from the ectodermal leaf. Furthermore, there are multiple feedback signals between the skin and the brain, which are mediated by inflammatory cytokines and neuro-immune pathways (Chen and Lyga 2014; Farzanfar et al. 2018).

There is a strong comorbidity between skin diseases and major depression (Dalgard et al. 2015; Marron et al. 2018) with exposure to stress affecting both the exacerbation of allergic reactions and depressive episodes (Niemeier et al. 2002). There is now evidence that depression is accompanied by activation of the immune-inflammatory response system (IRS) marked by increased levels of pro-inflammatory cytokines, such as interleukin (IL)-1, IL-6, tumor necrosis factor (TNF)-α, and interferon (IFN)-γ in the blood (Maes et al. 1991, 2009; Kubera et al. 2001, 2011b; Faugere et al. 2018; Dubois et al. 2018). Pro-inflammatory cytokines released in the periphery may cross the blood–brain barrier and affect microglia, which are probably involved in the pathophysiology of major depressive disorders (Troubat et al. 2020; Qin et al. 2007).

Antidepressants are used to treat major depression and these effects are in part explained by their immune-regulatory properties. In vitro studies revealed the dose-dependent ability of antidepressants to reduce the production of pro-inflammatory cytokines including TNF-α, IFN-γ, IL-6, and IL-1β by human lymphocytes and monocytes (Kubera et al. 2009; Xia et al. 1996). In animal models of depression, antidepressants reduce the production of IFN-γ and enhance the synthesis of IL-10 (Maes et al. 1999; Kubera et al. 2001).

Based on these findings, we have hypothesized that antidepressant drugs may have some efficacy in the treatment of contact hypersensitivity (CHS).

Our previous studies revealed that repeated administration of antidepressant drugs was effective in inhibiting CHS reactions to picryl chloride (PCL) in CBA/J mice and 2,4-dinitrofluorobenzene (DNFB) in Balb/c mice (Kubera et al. 2012; Curzytek et al. 2013). Fourteen-day treatment with antidepressants (10 days before immunization with a hapten and 4 days during this process) with both fluoxetine (a selective serotonin reuptake inhibitor, SSRI) and desipramine (a tricyclic antidepressant, TCA) used in two strains of mice inhibited CHS reactions by approximately 50%. Antidepressant treatment additionally reduced the production of the pro-inflammatory cytokines IL-6 and IFN-γ by concanavalin A (Con A)-stimulated splenocytes (Curzytek et al. 2013). Furthermore, the administration of both fluoxetine and desipramine during sensitization increased IL-10 production by Con A-stimulated splenocytes in a PCL model of CHS (Kubera et al. 2012) and by Con A-stimulated lymph node cells in a DNFB model of sensitization (Curzytek et al. 2013). Nazimek et al. (2016) showed that 6-day administration of imipramine, fluoxetine, and venlafaxine, before and during immunization with a hapten (PCL), attenuated CHS reactions in CBA/J mice by about 20%. These experimental investigations showed the effectiveness of antidepressants in inhibiting CHS responses. Nevertheless, the immune and molecular mechanisms underlying these anti-allergic effects remain unknown.

The present study aimed to investigate the mechanism of action of antidepressants in a cellular model of CHS using the activated human keratinocyte cell line HaCaT. Keratinocytes are the first cells involved in the response to chemicals contacting the skin and are, therefore, important in both the induction and elicitation phases of CHS responses. Due to the anatomical location of keratinocytes and their important role in inflammatory skin diseases, the use of keratinocytes in studies on the mechanisms underlying contact allergy is fully justified and commonly applied (Aye et al. 2020; Galbiati et al. 2011; Choi et al. 2013).

The aim of our study was to determine whether antidepressants have an anti-inflammatory effect on stimulated HaCaT cells. Toward this end, we investigated how antidepressants affect the secretion of cytokines, chemokines, cell adhesion molecule expression, and protein kinases by the stimulated keratinocytes HaCaT cell line.

## Materials and Methods

### Chemicals and Drugs

The following drugs were used: fluoxetine hydrochloride (Flu), desipramine hydrochloride (Des), and imipramine hydrochloride (Imi), all of which were obtained from Sigma-Aldrich, Germany. The following stimulants were used: lipopolysaccharide (LPS; 026:B6, Sigma-Aldrich, Germany), recombinant human TNF-α (Gibco, UK), recombinant human IFN-γ (Gibco, UK), and 1-fluoro-2,4-dinitrobenzene (DNFB; Sigma-Aldrich, Germany). The stock solutions of fluoxetine and DNFB were prepared in DMSO. A final concentration of the tested chemicals was prepared in distilled water, and the solvent was present in the cell culture at a final concentration of 0.01%. Desipramine and imipramine were dissolved in distilled water. LPS and TNF-α/IFN-γ were dissolved in the culture medium. Each experimental set of the control cultures was supplemented with the appropriate vehicle.

### Cell Culture

The human immortalized keratinocyte cell line (HaCaT) was purchased from CLS (Germany). The HaCaT cells were cultured in DMEM medium, supplemented with 10% FBS and 1% antibiotics (100 units/ml penicillin and 100 mg/ml streptomycin), and all reagents were obtained from Gibco, UK. The cells were cultured at 37 °C in a humidified atmosphere with 5% CO_2_. Cell cultures were carried out in 75 cm^2^ culture bottles. The cells were passaged once a week, in a volume ratio of 1:10. The cells were trypsinized with 0.05% Trypsin and 1 mM EDTA for 5 min at 37 °C from the bottom of the culture bottle. Cultures with a different number of cells were used for the tests, depending on the type of plates used and tests performed: 96-well plate — 5 × 10^4^ cells/well (for LDH release, MTT assay), 24-well plate — 5 × 10^5^ cells/well (enzyme-linked immunosorbent assay (ELISA) assay), and 6-well plate — 1 × 10^6^ cells/well (Western blot, quantitative real-time polymerase chain reaction (qRT-PCR)). On the day preceding the experiment, the culture medium was changed to DMEM with 1% FBS as well as penicillin/streptomycin antibiotics. The time of cell exposure to stimulants and antidepressants varied depending on the type of experiment. Drugs were added to cell cultures always 30 min before adding the appropriate stimulants. The control group was treated with a vehicle.

### Cell Viability Assay

The HaCaT cells were treated with various concentrations of antidepressants (Flu: 0.1–50 µM; Des: 0.1–10 µM, Imi: 0.1–10 µM) and stimulants (LPS: 0.5–10 µg/ml; TNF-α/IFN-γ: 1–50 ng/ml; DNFB: 0.01–50 µM) for 24 h. Subsequently, the MTT solution was added to each well (at a final concentration of 0.15 mg/ml) and incubated over 30 min at a temperature of 37 °C. The incubation was halted by the addition of 100% DMSO into each well, solubilizing the formazan. The absorbance at λ = 570 nm was measured using a microplate reader (Tecan, Infinite M200PRO). The data were normalized to vehicle-treated cells (100%) and expressed as a percent of control ± SEM established from at least 3 independent experiments with 5 replicates.

### LDH Release Assay

The amount of lactate dehydrogenase (LDH) released into culture media from HaCaT cells after 24 h of treatment with antidepressants and stimulants was measured with the Cytotoxicity Detection Kit (Roche). Briefly, part of the cell-free medium was removed, then the mixture of reagents supplied by the kit manufacturer was added and after 15 min of incubation at room temperature, absorbance at *λ* = 490 nm was measured using a microplate reader (Tecan, Infinite M200PRO). The data were normalized to vehicle-treated cells (100%) and expressed as a percent of control ± SEM established from at least 3 independent experiments with 5 replicates.

### Enzyme-Linked Immunosorbent Assay

The production of pro-inflammatory factors: IL-1β, IL-6, CXCL8 (IL-8), and CCL2 (MCP-1) was measured with the eBioscience ELISA Ready-SET-Go kit (USA) according to the manufacturer’s instructions. The level of each interleukin was measured in cell supernatants or cell lysates (IL-1β) from cell cultures, after 24 h of incubation with antidepressants and stimulants. The absorbance at *λ* = 450 nm was measured using a microplate reader (Tecan, Infinite M200PRO). The level of ICAM-1 was measured using the ELISA kit Peprotech (UK) and the level of E-cadherin was measured in cell lysates using the ELISA kit Life Technologies (USA), following the supplier’s recommendations.

### Western Blot

Western blot analysis was conducted to determine the level of kinase activation. Cell lysates were collected. One hour after the treatment of HaCaT cells with antidepressants (Flu 0.1 and 0.5 µM; Des 1 and 5 µM; Imi 1 µM) and stimulants (LPS: 3 µg/ml; TNF-α/IFN-γ: 10 ng/ml; DNFB: 1 µM), samples containing an equal amount of protein were separated by SDS–PAGE (4–20% gel; Bio-Rad, Hercules, CA, USA) and transferred to PVDF membranes (Trans-Blot Turbo; Bio-Rad, Hercules, CA, USA). After the transfer, the membranes were cut to allow simultaneous (overnight at 4 °C) incubation with different primary antibodies (anti-NFκB p65 (sc-372), anti-phospho-NFκB p65 (sc-33020), anti-IκB-α (sc-1643), anti-phospho-IκB-α (sc-8404), anti-NIK (sc-7211), anti-phospho-NIK (sc-12957), anti-p38 (sc-7972), and anti-phospho-p38 (sc-101759)) obtained from Santa Cruz Biotechnology (USA) and anti-GAPDH (MAB374, Millipore, USA). The next day, the membranes were washed four times with TBS containing 0.1% Tween-20 (TBST) and then incubated with appropriate secondary antibodies (Vector Laboratories, UK) for 1 h at room temperature. The immunoblots were visualized with a chemiluminescence detection kit (Roche, Germany). The data obtained were normalized to the level of reference proteins and then averaged and presented as a percentage of control ± SEM, from at least three independent experiments.

### Quantitative Real-Time Polymerase Chain Reaction

Total RNA was extracted from HaCaT cells using the Universal RNA Purification Kit (Eurx, Poland), according to the instructions provided by the manufacturer. Identical amounts of RNA (1 μg) were reverse transcribed into complementary DNA (cDNA) using a commercial RT-PCR kit (High-Capacity cDNA Reverse Transcription Kit, Applied Biosystems, USA). The cDNA was subsequently amplified using TaqMan probes and primers for the following genes: ICAM-1 (Hs00164932_m1) and E-cadherin (Hs01023894_m1) were obtained from the Life Technologies (USA), with the FastStart Universal Probe Master (Rox) kit (Roche, Basel, Switzerland). The threshold value (*C*_t_) for each sample was set in the exponential phase of PCR, and the ΔΔ*C*_t_ method was used for data analysis. Glyceraldehyde 3-phosphate dehydrogenase (GAPDH (Hs02758991_g1)) was used as the reference gene.

### Immunocytochemistry

HaCaT cells were seeded into LabTek II CC2 (Nunc, Thermo Fisher Scientific, USA) chambers coated with poly-o-lysine (0.01 mg/ml) at a density of 5 × 10^4^ per well. The cultures were grown for 48 h, then the culture medium was changed to 1% FBS serum content and then incubated with antidepressants (all drugs were used at the maximum concentrations) and stimulants (LPS and TNF-α/IFN-γ) for 1 h (anti-ICAM-1 conjugated to PE; orb124670, Biorbyt, UK) and for 24 h (anti-E-cadherin, conjugated to FITC; orb15536, Biorbyt, UK). Then, the cells were washed with PBS buffer and fixed with 4% paraformaldehyde in PBS for 10 min at room temperature. After three more washes with PBS buffer, the chambers were incubated for 10 min in an incubator at 95 °C, in the buffer revealing antigenic determinants (antigen retrieval buffer; 100 mM Tris, 5% urea, pH 9.5). Next, the chambers were again washed three times with PBS, and then the cells were blocked for 30 min in 1% BSA in PBST buffer (PBS + 0.1% Tween 20). The next step was a 1-h incubation with fluorochrome-labeled antibodies at room temperature in the dark. After the incubation, the chambers were again washed 3 times with PBS buffer. The plastic chambers were then removed from the slides and the stained slides were embedded in the VECTASHIELD® Antifade Mounting Medium with DAPI (Vector Laboratories, USA) and protected with a coverslip. Cells were visualized using a Leica TCS SP8 WLL confocal microscope, and image processing was performed using the Leica Application Suite X software (Leica, Germany).

### Statistical Analysis

The data were normalized and analyzed using the Statistica software, version 13 (StatSoft Inc., USA). Differences between group means were analyzed using a two-factor analysis of variance (factorial ANOVA) followed by Tukey’s post hoc analysis. The differences were considered significant at *p* < 0.05.

## Results

### The Cytotoxicity Assessment of Tested Drugs and Stimulants

The cytotoxicity of both antidepressant drugs and stimulants in HaCaT keratinocytes was determined using the MTT reduction test and the lactate dehydrogenase (LDH) release test (Table 1). After 24 h of incubation, fluoxetine (Flu) in the concentration range of 0.1 to 5 μM was not toxic to the cells. A decrease in viability of HaCaT cells in the MTT test was only observed when using 50 μM fluoxetine (*p* < 0.05), while a fivefold increase in the level of LDH released into the culture medium had already been observed at the concentration of 10 μM (*p* < 0.05) (Table 1). However, the lowest tested drug concentrations were selected for a further study due to the slightly elevated (but statistically insignificant) levels of LDH released into the medium at 1 μM fluoxetine and the results of pilot experiments that indicated its cytotoxic activity at 1 μM.

**Table 1:** The effect of the antidepressant drugs and stimulants on the viability of HaCaT cells

	% of cell viability	% of LDH release
Drugs		
Control	100.00 ± 2.09	100.00 ± 1.74
Fluoxetine (Flu) [µM] **0.1**	**97.61 ± 3.29**	**98.78 ± 1.32**
**0.5**	**96.87 ± 1.32**	**87.35 ± 1.53**
1	95.60 ± 1.81	108.69 ± 1.19
5	93.27 ± 2.30	91.46 ± 1.01
10	102.91 ± 3.06	523.55 ± 50.56*
50	5.00 ± 0.52*	557.80 ± 8.15*
Control	100.00 ± 1.23	100.00 ± 3.01
Desipramine (Des) [µM] 0.1	97.05 ± 1.40	87.34 ± 1.29
**1**	**99.95 ± 1.28**	**93.92 ± 3.21**
**5**	**93.91 ± 1.82**	**92.73 ± 2.28**
10	91.79 ± 1.24*	113.83 ± 3.76*
Control	100.00 ± 1.94	100.00 ± 1.86
Imipramine (Imi) [µM] 0.1	99.40 ± 1.91	88.02 ± 2.70
**1**	**94.70 ± 1.65**	**87.23 ± 2.80**
5	85.50 ± 2.35*	84.94 ± 3.85*
10	78.10 ± 2.70*	70.82 ± 3.17*

Stimulants		
Control	100.00 ± 2.00	100.06 ± 1.62
LPS [µg/ml] 0.5	97.50 ± 2.82	96.28 ± 2.18
1	96.17 ± 2.54	92.11 ± 1.65*
**3**	**96.60 ± 1.86**	**93.79 ± 1.47**
5	81.19 ± 2.51*	99.39 ± 3.08
10	80.32 ± 2.34*	111.69 ± 1.67*
Control	100.00 ± 1.10	100.00 ± 1.64
TNF-α/IFN-γ [ng/ml] 1	98.62 ± 1.20	102.16 ± 1.49
5	96.07 ± 1.46	96.55 ± 1.75
**10**	**95.36 ± 0.87**	**98.54 ± 1.55**
20	93.47 ± 2.24*	91.89 ± 2.18*
50	91.91 ± 2.19*	112.69 ± 1.81*
Control	100.00 ± 3.05	100.00 ± 7.84
DNFB [µM] 0.01	93.63 ± 5.17	80.40 ± 3.01
0.1	86.70 ± 2.61	62.01 ± 3.34
**1**	**88.89 ± 3.42**	**88.34 ± 7.38**
10	8.46 ± 1.96*	518.97 ± 45.58*
50	2.44 ± 0.20*	774.23 ± 14.32*

Drugs + Stimulants		
LPS 3 + Flu 0.1	102.77 ± 2.38	86.98 ± 2.99
LPS 3 + Flu 0.5	100.78 ± 3.06	91.46 ± 3.10
LPS 3 + Des 1	89.81 ± 7.13	97.65 ± 1.79
LPS 3 + Des 5	88.97 ± 2.36	106.09 ± 5.28
LPS 3 + Imi 1	99.50 ± 2.19	101.07 ± 2.71
TNF-α/IFN-γ 10 + Flu 0.1	96.56 ± 2.58	112.45 ± 3.44
TNF-α/IFN-γ 10 + Flu 0.5	91.43 ± 4.34	106.63 ± 5.12
TNF-α/IFN-γ 10 + Des 1	95.14 ± 2.87	112.86 ± 6.04
TNF-α/IFN-γ 10 + Des 5	87.96 ± 2.53	117.80 ± 7.66
TNF-α/IFN-γ 10 + Imi 1	91.35 ± 2.98	110.79 ± 7.83
DNFB 1 + Flu 0.1	95.83 ± 1.68	93.94 ± 6.52
DNFB 1 + Flu 0.5	92.98 ± 1.88	94.03 + 6.76
DNFB 1 + Des 1	93.84 ± 3.37	93.19 ± 5.31
DNFB 1 + Des 5	86.19 ± 2.51	106.65 ± 7.13
DNFB 1 + Imi 1	96.77. ± 2.67	94.87 ± 3.31

Cells were treated with the fluoxetine (0.1–50 µM), desipramine (0.1–10 µM), imipramine (0.1–10 µM), and LPS (0.5–10 µg/ml), TNF-α/IFN-γ (1–50 ng/ml), or DNFB (0.01–50 µM) for 24 h, after which cell viability (MTT assay) and the level of released LDH were measured. The bold concentration values of individual substances were selected for further experiments. The results in the groups with combined stimulation of the cells by drugs and stimulants were compared with the appropriate control group (vehicle-treated cells) as well as the corresponding groups treated by drugs or stimulants alone. Data are shown as the mean of % of control ± SEM from at least 3 separate experiments with 5 replicates each. *p < 0.05 vs. control cells

Desipramine (Des) was not toxic to cells over the concentration range of 0.1–5 µM. In contrast, at the concentration of 10 μM, it showed a toxic effect on the cells, observed as a significant decrease in viability in the MTT test, and a simultaneous increase in the level of released LDH (*p* < 0.05) (Table 1).

The incubation of cells with imipramine (Imi) did not affect cell viability in the 0.1–1 µM concentration range. A decrease in viability was observed in the range of 5–10 μM (*p* < 0.05), while neither concentration of imipramine resulted in an increase in LDH secretion into the medium (Table 1).

Based on these results, we selected two concentrations of fluoxetine (0.1 and 0.5 µM) and desipramine (1 and 5 µM), and 1 µM imipramine (Table 1) to examine the effects of antidepressants on the stimulated production of the biomarkers.

Using the above-mentioned tests, LPS was non-toxic to cells in the concentration range of 0.5–3 µg/ml (Table 1), TNF-α/IFN-γ in the range of 0.5–10 µg/ml (Table 1), and DNFB from 10 nM to 1 µM (Table 1). The maximum non-toxic stimulant concentrations for the cultures were selected for further experiments, namely LPS at 3 µg/ml, TNF-α/IFN-γ at 10 ng/ml, and DNFB at 1 µM (Table 1). Finally, the combination of stimulants and antidepressants did not significantly affect the MTT test or the LDH release (Table 1).

### Effects of Antidepressant Drugs on the Production of Cytokines and Chemokines in LPS-, TNF-α/IFN-γ-, or DNFB-stimulated HaCaT cells

**Fig. 1:**
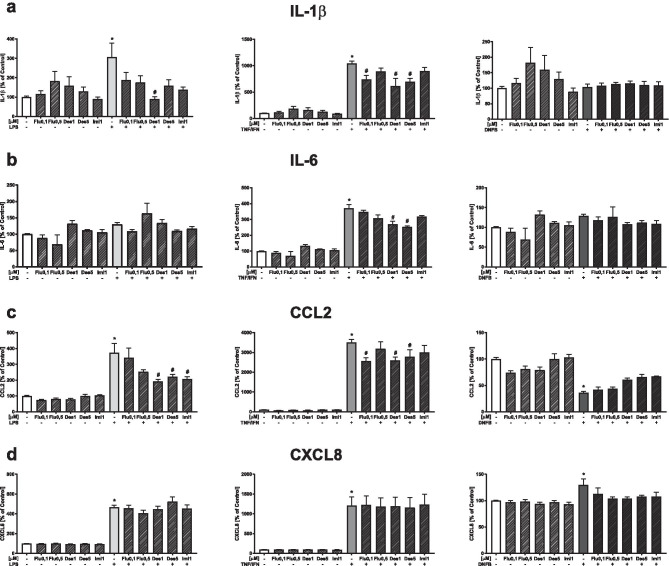
The effect of antidepressants on the level of IL-1β **a**, IL-6 **b**, CCL2 **c**, and CXCL8 **d** produced by HaCaT cells after stimulation with LPS (left side), TNF-α/IFN-γ (middle), and DNFB (right side). The level of cytokines was measured by ELISA after 24 h of incubation in medium from cell cultures as pg/ml (except IL-1β–lysates, measured in pg/mg protein). Data presented (mean ± SEM) as % control and are derived from three different experiments, where 3 wells of cell culture were used for each group. *p < 0.05 vs. control, #*p* < 0.05 vs. appropriate stimulated group. Flu, fluoxetine; Des, desipramine; Imi, imipramine; LPS, lipopolysaccharide; TNF/IFN, tumor necrosis factor (TNF)-α/interferon (IFN)-γ; DNFB, 2,4-dinitrofluorobenzene

LPS-treated HaCaT keratinocytes showed a threefold increase (*p* < 0.05) in IL-1β production. Pretreatment of HaCaT cells with antidepressants tended to reduce IL-1β secretion and only desipramine (1 μM, *p* < 0.05) significantly inhibited the production of IL-1β in LPS-stimulated cells (Fig. 1a).

When stimulating HaCaT cells with a TNF-α/IFN-γ, a tenfold increase in IL-1β secretion was observed (*p* < 0.05) and fluoxetine at 0.1 µM (*p* < 0.05) and desipramine at both concentrations (*p* < 0.05) reduced the level of secreted IL-1β (Fig. 1a).

Although we observed a tendency toward increased production of IL-6 in LPS-treated HaCaT cells, this change did not reach statistical significance. Furthermore, the antidepressants examined here did not affect this parameter (Fig. 1b).

Upon the TNF-α/IFN-γ stimulation of HaCaT, the cells produced three times more IL-6 (*p* < 0.05). Desipramine pretreatment for 30 min, at both 1 and 5 µM (*p* < 0.05), reduced the TNF-α/IFN-γ-evoked increase in the IL-6 production (Fig. 1b).

The stimulation of HaCaT cells using DNFB or antidepressants, as well as both factors simultaneously, did not lead to changes in the secretion of IL-6 into the medium (Fig. 1b).

LPS significantly upregulated CCL2 (MCP-1) in the HaCaT culture medium (*p* < 0.05) in comparison to vehicle-treated cells. Moreover, desipramine at both concentrations (1 and 5 µM, *p* < 0.05) and imipramine (*p* < 0.05) significantly weakened the effect of LPS (Fig. 1c).

We observed that 24 h of TNF-α/IFN-γ stimulation upregulated the release of CCL2 up to 35-fold (*p* < 0.05) when compared to vehicle-treated cells. Furthermore, we observed that desipramine at concentrations 1 and 5 µM (*p* < 0.05) and fluoxetine at a lower dose (*p* < 0.05) effectively decreased the TNF-α/IFN-γ-evoked upregulation in the chemokine secretion (Fig. 1c).

DNFB stimulation significantly decreased the production of CCL2 by HaCaT cells (*p* < 0.05) and antidepressants did not have a significant effect on the CCL2 production (Fig. 1c).

 The secretion of CXCL8 was significantly elevated by all stimulators of HaCaT cells (*p* < 0.05). TNF-α/IFN-γ had the strongest stimulatory effect followed by LPS, while there was a slight increase after administration of DNFB. Antidepressants did not affect the normalization of this parameter after administration of the stimulatory factors (Fig. 1d).

There was no effect of the antidepressant drugs under basal conditions on either of the investigated pro-inflammatory factors (Fig. 1a–d).

### Effects of Antidepressant Drugs and LPS, TNF-α/IFN-γ, or DNFB Stimulation on ICAM-1 Expression in HaCaT Cells

ICAM-1 protein expression level was determined with ELISA in lysates of HaCaT cells (24 h of incubation), and the expression level of mRNA using real-time PCR (4 h of incubation) in both vehicle-treated control cultures and stimulant-treated cultures.

**Fig. 2:**
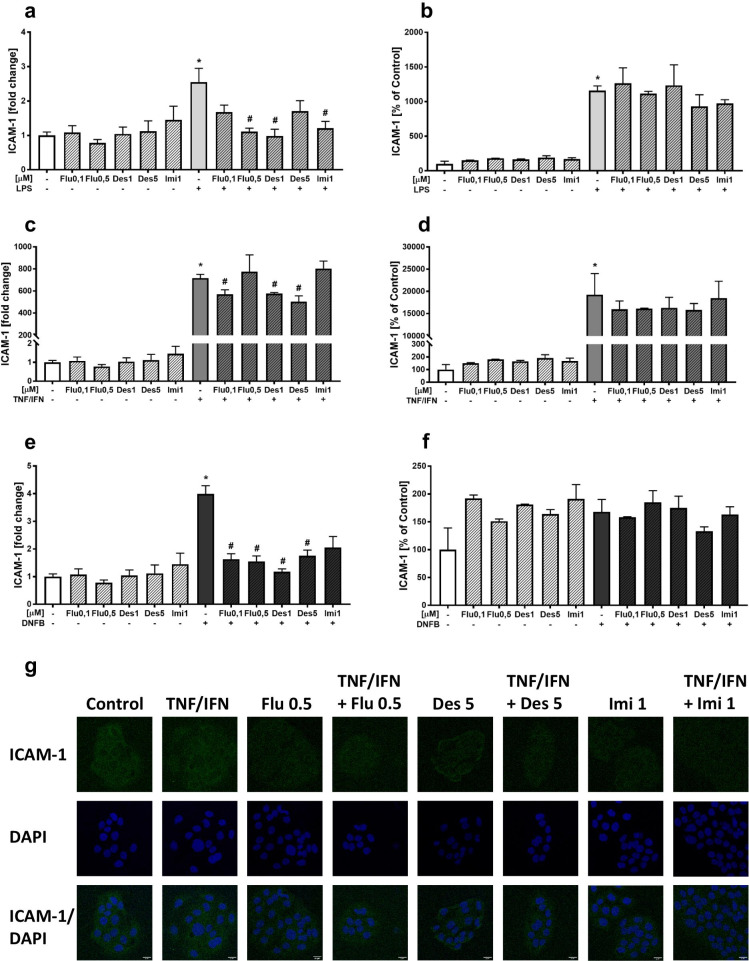
The effect of antidepressants on the gene expression of ICAM-1 (left panel; **a**, **c**, **e**) and the protein level of ICAM-1 (right panel; **b**, **d**, **f**) in HaCaT cells after stimulation with LPS, TNF-α/IFN-γ, and DNFB. The expression of ICAM-1 in cell lysates was determined after 4 h of incubation, and the results are presented as the mean fold change (± SEM) relative to the reference gene (GAPDH). ICAM-1 protein level was assessed by ELISA after 24 h incubation; in cell culture lysates, the measurement was done in pg/mg protein, the averaged results are presented as % control (± SEM). The presented data come from three different experiments (for individual types of assays), where for each group, there were 3 wells of cell culture. *p < 0.05 vs. control, #*p* < 0.05 vs. appropriate stimulated group. Representative fluorescence photomicrographs **g** showing the effect of TNF-α/IFN-γ and antidepressants: fluoxetine (Flu, 0.5 µM), desipramine (Des, 5 µM), or imipramine (Imi, 1 µM) on ICAM-1 expression in HaCaT cells, incubated for 1 h. The ICAM-1 signal was immunodetected using anti-human, staining with anti-CD54 PE (ICAM-1) antibody, and nuclei were stained using DAPI labeling. Scale bar (20 μm) is located in the bottom right corner of each image. Flu, fluoxetine; Des, desipramine; Imi, imipramine; LPS, lipopolysaccharide; TNF/IFN, tumor necrosis factor (TNF)-α/interferon (IFN)-γ; DNFB, 2,4-dinitrofluorobenzene

We observed a stimulatory effect of LPS on the ICAM-1 gene expression in HaCaT cells (*p* < 0.05) and found significantly decreased ICAM-1 mRNA expression after pretreatment with fluoxetine (0.5 µM, *p* < 0.05), desipramine (1 µM, *p* < 0.05), and imipramine (1 µM, *p* < 0.05) (Fig. 2a). After the stimulation of the cells with LPS, an increase in ICAM-1 protein expression was observed (*p* < 0.05), although the antidepressants used herein did not significantly decrease ICAM-1 (Fig. 2b).

In the case of the TNF-α/IFN-γ stimulation, keratinocytes dramatically enhanced the expression of ICAM-1 mRNA (*p* < 0.05). Furthermore, fluoxetine in a higher concentration (0.5 µM, *p* < 0.05) and desipramine at both 1 and 5 µM (*p* < 0.05) reduced the stimulated mRNA expression of ICAM-1 (Fig. 2c). ICAM-1 protein expression was significantly elevated after the stimulation with TNF-α/IFN-γ (*p* < 0.05) and pretreatment with antidepressant drugs did not significantly affect the stimulated ICAM-1 protein expression (Fig. 2d). These results were also replicated using confocal microscopy after staining the HaCaT cells with an anti-ICAM-1 antibody (1 h of incubation) (Fig. 2g).

The stimulation of HaCaT cells with DNFB increased the expression of ICAM-1 (*p* < 0.05). Furthermore, we demonstrated that fluoxetine (0.1 and 0.5 µM, *p* < 0.05) and desipramine (1 and 5 µM, *p* < 0.05) pretreatment effectively decreased the DNFB-induced upregulation of ICAM-1 mRNA levels (Fig. 2e). After DNFB stimulation, there was no significant effect of antidepressants on ICAM-1 protein levels (Fig. 2f).

### Effects of Antidepressant Drugs and LPS, TNF-α/IFN-γ, or DNFB Stimulation on the E-Cadherin Expression in HaCaT Cells

**Fig. 3:**
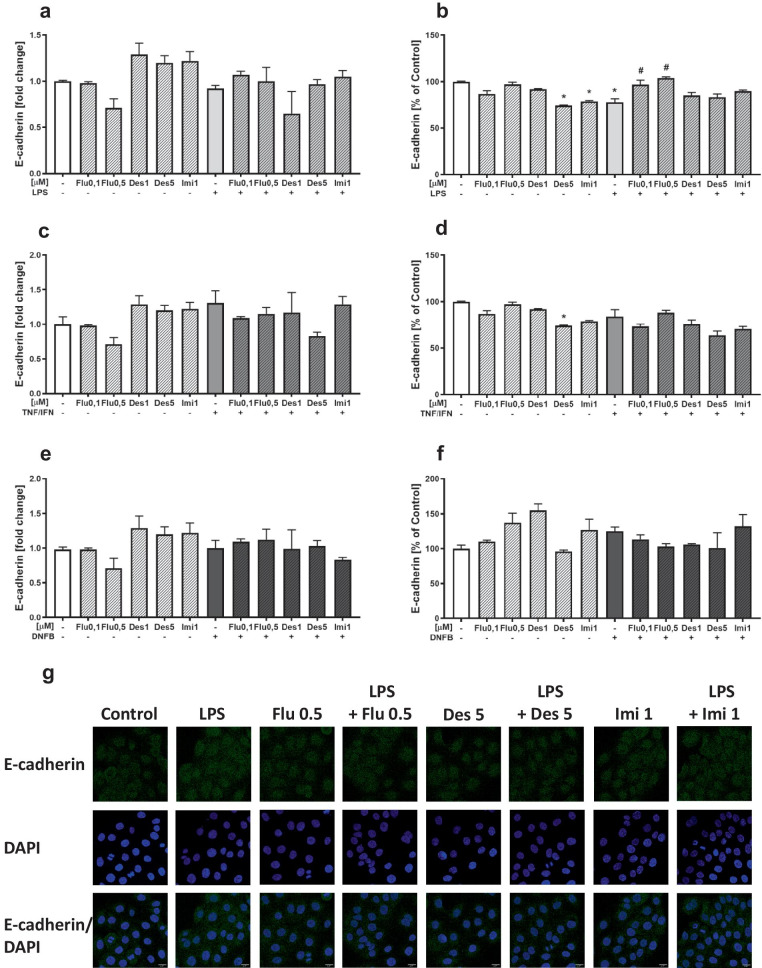
The effect of antidepressants on the gene expression of E-cadherin (left panel; **a**, **c**, **e**) and the protein level of E-cadherin (right panel; **b**, **d**, **f**) in HaCaT cells after stimulation with LPS, TNF-α/IFN-γ, and DNFB. The expression of E-cadherin in cell lysates was determined after 4 h of incubation, and the results are presented as the mean fold change (± SEM) relative to the reference gene (GAPDH). E-cadherin protein level was assessed by ELISA after 24 h of incubation; in cell culture lysates, the measurement was done in pg/mg protein, the averaged results are presented as % of control (± SEM). The presented data come from three independent experiments (for individual types of assays), where for each group, there were 3 wells of cell culture. *p < 0.05 vs. control, #*p* < 0.05 vs. LPS-stimulated group. Representative fluorescence photomicrographs (**g**) showing the effect of LPS and antidepressants: fluoxetine (Flu, 0.5 µM), desipramine (Des, 5 µM), or imipramine (Imi, 1 µM) on E-cadherin expression in HaCaT cells, incubated for 24 h. The E-cadherin signal was immunodetected using anti-human, staining with anti-E-cadherin FITC antibody, and nuclei were stained using DAPI labeling. The scale bar (20 μm) is located in the bottom right corner of each image. Flu, fluoxetine; Des, desipramine; Imi, imipramine; LPS, lipopolysaccharide; TNF/IFN, tumor necrosis factor (TNF)-α/interferon (IFN)-γ; DNFB, 2,4-dinitrofluorobenzene

In our study, both the 4-h incubation of keratinocytes with LPS and antidepressants had no effect on E-cadherin gene expression (Fig. 3a). LPS stimulation of cells decreased E-cadherin protein expression and fluoxetine (0.1 and 0.5 µM, *p* < 0.05) normalized the effects of LPS. We also observed a decrease in E-cadherin protein expression after treatment with desipramine (5 µM, *p* < 0.05) and imipramine (1 µM, *p* < 0.05) (Fig. 3b).

The effects of LPS stimulation and antidepressant drugs on the expression of E-cadherin on HaCaT keratinocytes were qualitatively confirmed by immunocytochemical staining using confocal microscopy (Fig. 3g).

There was no effect of the TNF-α/IFN-γ stimulation (Fig. 3c, d) or DNFB stimulation (Fig. 3e, f) of HaCaT cells on E-cadherin levels, and both gene and protein expression. Pretreatment with antidepressants did not affect E-cadherin in stimulated cells.

### Effects of Antidepressant Drugs and LPS, TNF-α/IFN-γ, or DNFB Stimulation on the Activation of NF-κB and p-38 Pathway in HaCaT Cells

**Fig. 4:**
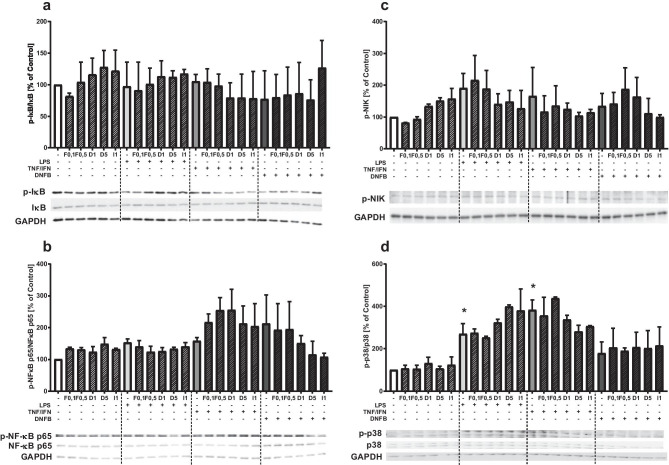
The effect of antidepressants and stimulation on the level of phosphorylation of proteins: Iκb **a**, p65 NF-κB , NIK **c**, and p38 **d** in HaCaT cells. Protein levels were assessed by Western blotting in samples incubated for 1 h. Histograms show band density normalized to the reference protein, averaged (from 3 different experiments), and presented as % of control ± SEM, with representative immunoblots. *p < 0.05 vs. control group. F, fluoxetine; D, desipramine; I, imipramine; LPS, lipopolysaccharide; TNF/IFN, tumor necrosis factor (TNF)-α/interferon (IFN)-γ; DNFB, 2,4-dinitrofluorobenzene

To further investigate the intracellular mechanism underlying the anti-inflammatory effects of antidepressants in the stimulated HaCaT cell line, we assayed kinase activation. We found that LPS or TNF-α/IFN-γ stimulation for 1 h activated p38 kinase. Pretreatment with antidepressant drugs showed no effect on the stimulant-evoked increase in phosphorylation of p38 kinase; however, under the influence of desipramine and imipramine, we observed a tendency to decrease the level of p38 in cultures stimulated with TNF-α/IFN-γ and an increase in the level of the phosphorylated form of kinase p38 in LPS-stimulated cultures (Fig. 4d).

We observed a tendency to increase NIK kinase activation under the influence of LPS or TNF-α/IFN-γ and a tendency to inhibit NIK kinase activation after using antidepressant drugs in a cell culture (Fig. 4c). Our results indicate that both under the influence of stimulants and various antidepressants, there are no significant effects on the phosphorylated form of IκBα (kappa B inhibitor) (Fig. 4a).

The use of DNFB in HaCaT keratinocyte cultures caused an increase in the level of the active form of the NF-κB p65 subunit, and tricyclic antidepressants tended to reduce the level of p-NFκB p65. LPS and TNF-α/IFN-γ caused less phosphorylation of NF-κB p65 and the use of antidepressants did not affect NF-κB p65 expression (Fig. 4b).

## Discussion

The first major finding of this study is that antidepressants suppress some aspects of the inflammatory response in HaCaT keratinocytes. Imipramine, desipramine, and fluoxetine have beneficial anti-inflammatory effects as indicated by the diminished production of inflammatory factors and suppression of the inflammatory response. We found that after stimulation with LPS or TNF-α/IFN-γ, HaCaT keratinocytes synthesize significantly more IL-1β than unstimulated cells and that antidepressants significantly decrease IL-1β production in activated HaCaT cells. The IL-1 family plays a leading role in the induction and elicitation of contact hypersensitivity in both animal models and humans as well (Mattii et al. 2013; Enk and Katz 1995). In mice, the application of the skin allergen increased the expression of IL-1β mRNA (Kermani et al. 2000). Furthermore, biopsy skin fragments from patients with ACD showed elevated IL-1β, IL-33, IL-36α, IL-36β, and IL-36γ mRNA expression (Mattii et al. 2013).

Previously, researchers observed that imipramine, clomipramine, and citalopram reduce IL-1β secretion by mitogen-stimulated monocytes (Xia et al. 1996). Furthermore, imipramine, fluoxetine (Obuchowicz et al. 2014), and tianeptine (Ślusarczyk et al. 2018) decrease IL-1β mRNA expression (also IL-18) and IL-1β production in LPS-stimulated microglia cultures. Dao-Ung et al. (2015) showed that paroxetine blocks the human P2X7 receptor, which is activated during immunization with a hapten, leading to activation of nucleotide-binding oligomerization domain-like (NOD-like) receptor pyrin-containing 3 inflammasome (NLRP3) inflammasome subunits and production of mature forms of IL-1β and IL-18 (Martin et al. 2008; Silvestre et al. 2018). We also reported that antidepressant drugs may reduce IL-1β release from mitogen-stimulated spleen cells isolated from rats under the chronic mild stress procedure (Kubera et al. 1996). However, recent meta-analyses have shown no effects of antidepressants on IL-1β levels in major depressed (MDD) patients (Köhler et al. 2018; Liu et al. 2020).

We also found that desipramine had an inhibitory effect on the release of IL-6 in TNF-α/IFN-γ-stimulated cells, whereas fluoxetine did not have such effects on TNF-α/IFN-γ/DNFB-stimulated cells and even stimulated IL-6 in LPS-stimulated keratinocytes. There is now evidence that IL-6 is elevated in MDD patients, although antidepressants may increase (Maes et al. 1997; Kubera et al. 2000, 2004; Fornaro et al. 2011) or decrease (Xia et al. 1996; Himmerich et al. 2010; Köhler et al. 2018; Duda et al. 2019) serum IL-6 levels. Previous studies in animals showed that fluoxetine and desipramine treatment increased the secretion of IL-6 by splenocytes in response to mitogen stimulation (Curzytek et al. 2013). However, the use of both drugs in animals subjected to contact sensitization resulted in a decrease in its secretion by spleen cells. In addition, the level of IL-6 release was not regulated by antidepressants and/or CHS in stimulated lymph node cells (Curzytek et al. 2013) or splenocytes (Kubera et al. 2012). The increase in IL-6 production by keratinocytes may be a compensatory response as indicated, for example, by a significant inverse association between increased IL-6 and decreased TNF-α, cytokines that are activated in the early phase of CHS (Flint et al. 1998). In addition, recent research indicates that the anti-inflammatory potential of IL-6 may proceed through the membrane IL-6Rα receptor, which is present in keratinocytes (Frempah et al. 2019).

The second major finding of this study is that stimulated HaCaT keratinocytes exhibit an increased production of chemokines and that antidepressants may modulate some but not all chemokine levels. First, activation with LPS or TNF-α/IFN-γ increased the synthesis of CCL2 (monocyte chemoattractant protein-1; MCP-1), and fluoxetine, desipramine, and imipramine decreased the stimulated production of CCL2. CCL2 plays a leading role in the elicitation phase of CHS, through increased production by skin residing cells (keratinocytes, fibroblasts, endothelial cells), followed by infiltration of monocytes and lymphocytes. This chemokine appears rapidly and its high concentrations persist for many hours after exposure to a hapten (Goebeler et al. 2001). Transgenic mice overexpressing CCL2 react to immunization with a DNFB-enhanced inflammatory response and greater infiltration of dendritic cells into the skin. Importantly, lowering the level of this chemokine resulted in the attenuation of the CHS-induced reaction (Mizumoto et al. 2001). Furthermore, overexpression of CCL2 in the course of CHS in mice caused an increase in itching and pain that accompany this disease (Jiang et al. 2019). Few studies reported on the impact of antidepressants on chemokine levels. For example, in a murine model of sepsis, amitriptyline decreased the serum levels of CCL2 and CXCL1 (KC, keratinocyte-derived chemokine) (Xia et al. 2019). In the brain of prenatally stressed rats, the therapeutic potential of antidepressants, including tianeptine, venlafaxine, and fluoxetine, is associated with regulatory activities targeted at CXCL12, and CX3CL1 and their CX3CR1, CXCR4, and CXCR7 receptors (Trojan et al. 2017). Moreover, tianeptine administration may reduce the level of CCL2 in LPS-stimulated microglia cultures (Ślusarczyk et al. 2018). O’Sullivan et al. (2010) observed that single intraperitoneal administrations of desipramine and atomoxetine (both noradrenaline reuptake inhibitors) to rats inhibited the LPS-increased expression of CCL5 (RANTES, regulated on activation, normal T cell expressed and secreted) and CXC10 (IP-10) mRNA in the frontal cortex, hippocampus, and spleen. Other authors showed that atomoxetine and desipramine increased CCL2 secretion in primary astrocyte cultures (Hinojosa et al. 2011). In MDD patients, treatment with antidepressants decreased (Köhler et al. 2018) or did not affect serum CCL2 levels (Ho et al. 2017).

However, chemokines exert a Janus-faced activity whereby initial increased concentrations stimulate the influx of macrophages and monocytes toward the site of inflammation, whereas in a later stage, the same chemokines may drive the influx of T regulatory cells which may inhibit the hypersensitivity reaction. Therefore, blocking the release of these chemokines may attenuate both inflammatory and regulatory mechanisms. Interestingly, the use of DNFB alone inhibits CCL2 secretion in keratinocytes and the antidepressants do not normalize this effect. Other authors, who investigated the effects of DNCB or TNCB on CCL2 gene expression in the skin of mice, did not observe any differences in the level of CCL2 mRNA in the sensitized mice group compared to the control groups (Chen et al. 2020; Gautam et al. 1994). Haptens increased mRNA synthesis of CCL2 in fibroblast cells and the extracellular matrix of dermal collagen (Gautam et al. 1994). Perhaps the presence of DNFB inhibits the CCL2 O-glycosylation process in HaCaT cells which shortens the half-life of the CCL2 protein (hence the decrease in CCL2 levels observed after 24 h of DNFB stimulation) but increases the bioactivity of this chemokine (Ruggiero et al. 2003).

We observed that keratinocyte stimulants increased the secretion of CXCL8 (IL-8) and that antidepressants did not reduce CXCL8 secretion by stimulated keratinocytes. During contact sensitization to picryl chloride in mice, an increase in the synthesis of CXCL1, CXCL2, and CXCL5 (homologous to CXCL8) was observed (Sakai et al. 2019), whereas nickel allergy was associated with a decrease in CXCL8 in humans (Summer et al. 2018). In patients suffering from MDD, antidepressants did not affect peripheral levels of CXCL8 (Köhler et al. 2018; Liu et al. 2020).

The third important finding of this study is that various stimulants (especially TNF-α/IFN-γ) may increase the production of ICAM-1 in HaCaT cells and that antidepressants decrease ICAM-1 mRNA expression in stimulated cell cultures. ICAM-1 plays a key role in CHS because a lack of ICAM-1 inhibits DNFB-induced CHS (Ogawa et al. 2010). Likewise, the use of anti-ICAM-1 or anti-LFA-1 (ICAM-1 ligand) antibodies inhibits the rolling process and migration of T lymphocytes to local lymph nodes during an ongoing inflammatory response (Teijeira et al. 2017). Moreover, tolerance to low doses of allergens applied to the skin of mice was associated with the abolition of elevated ICAM-1 expression in the effector phase of the CHS reaction (Komura et al. 2009). We observed that all antidepressants lowered ICAM-1 gene expression. Other researchers observed that a single, intraperitoneal administration of atomoxetine or desipramine to LPS-treated Sprague Dawley rats inhibited LPS-induced ICAM-1 mRNA expression in the frontal cortex, hippocampus, and spleen of experimental animals (O’Sullivan et al. 2010). Mirtazapine administered in a mouse model of autoimmune hepatitis induced a decrease in the levels of many pro-inflammatory factors, including ICAM-1 in the liver (Almishri et al. 2019). Fluoxetine, administered for 3 weeks to rats with induced pulmonary arterial hypertension, decreased ICAM-1 protein expression in lung tissues (Li et al. 2011). The opposite effect was observed in a fluoxetine-treated (for 4 weeks) mouse model of atherosclerosis, which showed an increased expression of ICAM-1 on neutrophils and monocytes, but not endothelial cells (Rami et al. 2018).

We found that antidepressants also modulate the expression of E-cadherin in stimulated HaCaT cell cultures. These antidepressants did not affect E-cadherin gene expression, whereas desipramine and imipramine reduced E-cadherin protein levels and only fluoxetine normalized the LPS-reduced expression of this adhesive molecule. This normalizing effect of fluoxetine on the impaired expression of E-cadherin may suggest that this drug has efficacy in maintaining integrity or tissue homeostasis. On the other hand, the reduced expression of E-cadherin may be associated with the weakening of the skin barrier, thereby promoting allergic reactions, a phenomenon which is occasionally observed in patients exposed to antidepressants (Beer 1994; Sanz-Gallén et al. 2011; Doffoel-Hantz et al. 2009; Gillet-Terver et al. 1996; Lin et al. 2009). Adverse effects of fluoxetine were also observed in pancreatic β cells, with incubation of murine cell line MIN6 with fluoxetine being accompanied by impairment of cell function (Chang et al. 2017). Moreover, the reduction in E-cadherin levels on tumor cells is associated with the formation of metastases (Cavallaro and Christofori 2001). In our previous studies, we showed that desipramine and fluoxetine significantly increase the likelihood of metastases to the spleen, liver, skin, gastrointestinal tract, and peritoneal cavity in mice with skin melanoma induced by B16F10 tumor cell implantation (Kubera et al. 2011a).

Regulation of expression of adhesion molecule ICAM-1 plays an important role in both the induction and elicitation of contact hypersensitivity response. Under physiological conditions, the increased expression of ICAM-1 facilitates the rolling, adhesion, and endothelial transmigration of effector T cells to sites of inflammation and therefore influences the magnitude of the inflammatory process at the site (Harjunpää et al. 2019). Cadherins represent a large family of glycoproteins associated with the cell membrane. They are involved in the calcium-dependent regulation of cell adhesion, regulate the morphogenesis of tissue, and are responsible for the maintenance of tissue continuity and coordination of the movement of the cells (Van den Bossche and Van Ginderachter 2013). The expression of E-cadherin on the surface of keratinocytes not only allows to maintain the tight junctions between the cells forming the epidermis but also constitutes a “mechanical trap” for the resident dendritic cells in the skin. Decreased expression of E-cadherin on the surface of dendritic cells and keratinocytes under the influence of inflammatory factors is necessary for the migration of dendritic cells into the lymph nodes (Brand et al. 2019). Proper cell adhesion is essential for maintaining homeostasis and correct functioning of cells and tissues. Therefore, our findings showing that antidepressants may normalize the expression of ICAM-1 and E-cadherin in HaCaT keratinocytes may partly explain their anti-inflammatory action.

The effectiveness of antidepressants in inhibiting ICAM-1 was observed in mRNA expression, but not in the protein level. This difference may be due to different kinetics of expression of the ICAM-1 gene and protein. Likewise, in endothelial cells stimulated with IL-1β, IFN-γ, and TNF-α, the maximum ICAM-1 mRNA expression was observed after 4 h of incubation and a decrease in expression after 7 h, whereas ICAM-1 protein levels were continuously increased in the stimulated cell culture, 1 to 24 h later (Barton et al. 1995). HaCaT cell stimulation led to a tremendous increase in ICAM-1 mRNA, and the inhibitory effect of antidepressants on ICAM-1 gene expression was observed at the time of maximum expression. We did not observe a statistically significant inhibitory effect of antidepressants on the increase of ICAM-1 protein levels stimulated with LPS or TNF-α/IFN-γ, despite the decrease in mRNA expression (Fig. 2).

Interestingly, patients with depression have increased ICAM-1 levels in serum (Dimopoulos et al. 2006) and the dorsolateral prefrontal cortex (Thomas et al. 2000). Moreover, it is suggested that antidepressant efficacy may be explained by regulating neuronal cell adhesion molecule (NCAM) (Wedzony et al. 2013). Such effects may also explain that antidepressants may have inhibitory effects on contact hypersensitivity in animal models, as we previously investigated (Kubera et al. 2012; Curzytek et al. 2013, 2015).

The fourth finding of this study is that the stimulation of LPS and TNF-α/IFN-γ activated p38 kinase while there were no significant effects on the other signaling pathways assayed here. There were no significant effects of antidepressants and only trends toward significant effects were observed. Few studies show that contact hypersensitivity reactions are accompanied by NF-κB and MAP kinase activation in keratinocytes, dendritic cells, and T cells (Bell et al. 2003; Ritprajak et al. 2012; Galbiati et al. 2011; Honda et al. 2013; Martin et al. 2008). Antidepressant drugs decrease the activation of NF-κB and MAP kinases in animal models of depression (Obuchowicz et al. 2014; Yang et al. 2014; Roumestan et al. 2007), although there are also negative reports (Martín-Hernández et al. 2018). Previous data indicate that the anti-inflammatory effects of antidepressants are at least in part attributable to their ability to regulate the p38 kinase signal transduction pathway whereby the activation of p38 is associated with increased expression of the serotonin transporter, which is targeted by antidepressants (Haroon et al. 2012).

HaCaT cells also express antidepressant metabolizing enzymes, although the metabolism of drugs in the skin is about 300-fold lower than in the liver (Kazem et al. 2019). However, it cannot be excluded that the anti-inflammatory effect of antidepressants on the inflamed HaCaT cell line may be explained by active drug metabolites.

In summary, the antidepressant drugs examined in our study effectively inhibited the stimulated secretion of pro-inflammatory cytokines, chemokines, and modulated the expression of adhesion molecules by the keratinocyte cell line HaCaT. Therefore, antidepressants may have some clinical efficacy in patients with ACD and especially in patients with comorbid depression and contact allergy. Our results show the beneficial role of antidepressants in ameliorating the pro-inflammatory responses in keratinocytes and the ability of antidepressant drugs to reduce peripheral inflammation induced by inflamed keratinocytes. The results of our study show that keratinocytes may contribute to the general immune-inflammatory response and, consequently, may affect neuroinflammation and microglial activation in the central nervous system.
